# Infiltration of Bilateral Optic Nerves in Burkitt Lymphoma: A Case Report

**DOI:** 10.7759/cureus.25515

**Published:** 2022-05-31

**Authors:** Ng Kwang Sheng, Wan-Hazabbah Wan Hitam, Nurul Ain Masnon

**Affiliations:** 1 Department of Ophthalmology and Visual Science, School of Medical Sciences, Universiti Sains Malaysia, Kelantan, MYS; 2 Ophthalmology Clinic, Hospital Universiti Sains Malaysia, Universiti Sains Malaysia, Kelantan, MYS; 3 Department of Ophthalmology, Hospital Kuala Lumpur, Kuala Lumpur, MYS

**Keywords:** cns prophylaxis, imaging, ocular manifestations, optic nerve infiltration, burkitt lymphoma

## Abstract

Burkitt lymphoma (BL) is one of the highly aggressive non-Hodgkin B-cell lymphomas. The optic nerve can be affected in case of isolated lymphoma or together with the central nervous system (CNS) and systemic lymphoma. We report a rare case of involvement of bilateral optic nerves in BL. A 31-year-old lady who was diagnosed with BL presented with severe intermittent headache and vomiting with blurring of vision in both eyes for one week. Visual acuity on presentation was 6/9 in the right eye and 6/24 in the left eye, with a reduction of the left eye optic nerve functions. Fundoscopy showed swollen optic disc in the right eye and temporal pallor disc in the left. Magnetic resonance imaging of the brain and orbit showed increased leptomeningeal enhancement in the right frontal and temporal lobes and the right optic nerve. Lumbar puncture revealed high opening pressure (50 cmH_2_O). Pleocytosis and the presence of lymphomatous infiltration were noted in cerebrospinal fluid analysis. After the completion of four cycles of chemotherapy, her condition unfortunately deteriorated, and she was subsequently planned for palliative therapy. CNS-directed therapies should be considered given the high risk of CNS relapse.

## Introduction

Lymphoma is the fourth most common cancer in Malaysia with an incidence of 5.1%, after breast, colorectal, and lung cancers [1]. The lifetime risk for males is 1 in 176 and 1 in 252 for females. It is the most common cancer in the 15-24-year age groups in both genders. Lymphoma is divided into two major categories, namely, Hodgkin’s lymphoma (HL) and non-Hodgkin’s lymphoma (NHL). HL is characterized by Reed-Sternberg (HRS) cells, while NHL is further subdivided into T-cell NHL (15%) and B-cell NHL (85%). Overall, 60% of B-cell NHLs fall into the aggressive category. Burkitt lymphoma (BL) is a high-grade, aggressive type of B-cell NHL. Involvement of the retina, vitreous, and optic nerve head can be up to 20% in primary central nervous system (CNS) lymphoma cases [2], but the optic nerve infiltration by B-cell NHL is rare [2,3]. We report a rare case of a 31-year-old lady with BL who developed simultaneous bilateral optic nerve and CNS infiltration. We also discuss the literature review relevant to this case.

## Case presentation

A 31-year-old lady with underlying BL presented with intermittent headache and vomiting for one week. It was followed by the blurring of vision in both eyes but mainly the left. The blurring of vision was generalized and progressively worsened. There was no diplopia, redness, photophobia, floaters, or pain on eye movement. She was diagnosed with stage IV BL four months ago when she presented with persistent headaches. A bone marrow biopsy confirmed the diagnosis. She received three cycles of chemotherapy (regime of dose-adjusted etoposide, prednisone, vincristine, cyclophosphamide, doxorubicin, and rituximab, DA-EPOCH-R).

**Figure 1:**
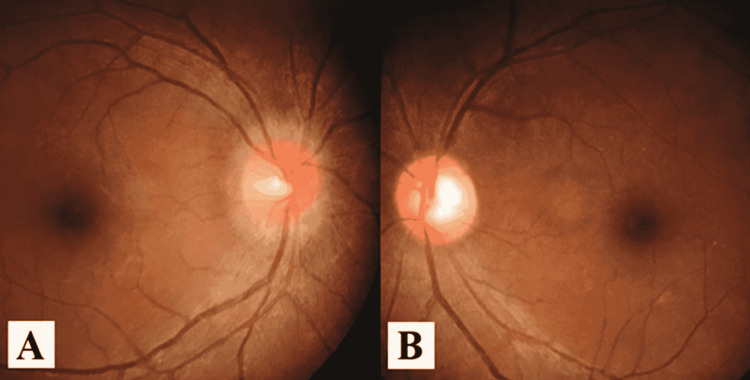
Fundus photo shows (A) hyperemic and swollen right disc, and (B) temporal disc pallor of the left eye.

On examination, the visual acuity in the right eye was 6/9 and 6/24 in the left eye. The relative afferent pupillary defect was positive in the left eye. Other optic nerve functions including color vision, light brightness, and red saturation were affected in the left eye. The confrontation test was normal. There was no proptosis, ocular deviation, or limitation of extraocular muscle movements. Both anterior segments and intraocular pressure were normal. Fundoscopy revealed the right hyperemic swollen disc. There was no disc hemorrhage, infiltration, or exudates. The left disc showed temporal pallor (Figure 1). The vitreous was clear and there was no evidence of posterior uveitis. Examinations of other cranial nerves were normal. The neurological examination was unremarkable. Systemic examination revealed that the patient was in pain, and vital signs were stable. No abdominal mass or lymphadenopathy was noted.

**Figure 2:**
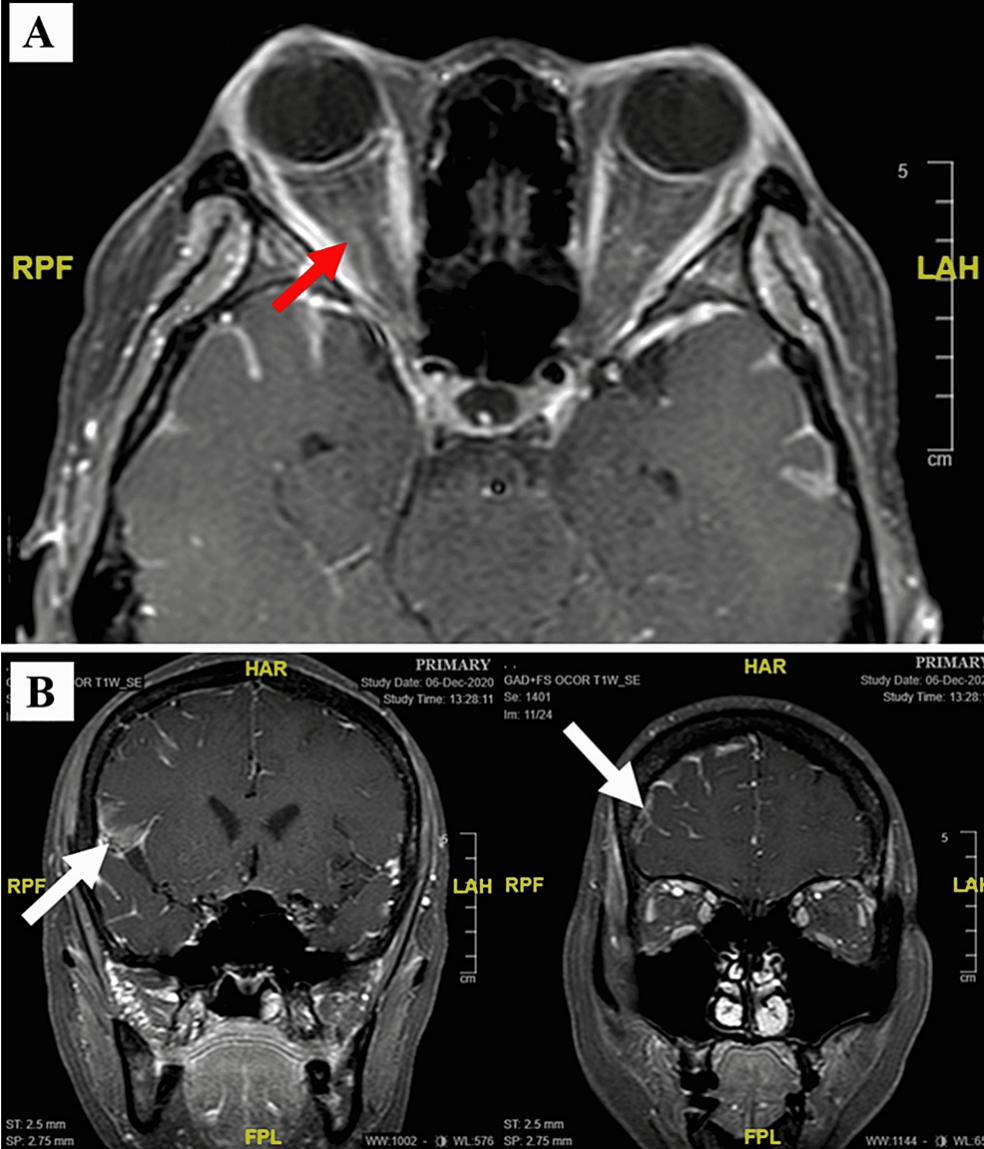
(A) Contrast enhancement of the right optic nerve (red arrow) in gadolinium-enhanced T1-weighted MRI. (B) MRI showing leptomeningeal enhancement (white arrows). MRI: magnetic resonance imaging

Magnetic resonance imaging (MRI) showed enhancement of the right optic nerve with the leptomeningeal enhancement in the right frontal and temporal lobe (Figure 2). There was no mass seen to support Foster-Kennedy syndrome. The opening pressure during lumbar puncture was high at 50 cmH_2_O. The cerebrospinal fluid (CSF) analysis showed pleocytosis and the presence of lymphomatous infiltration (lymphocytes of 60 mm^3^). The infective screening was negative for human immunodeficiency virus, syphilis, tuberculosis, and cryptococcosis. She received one cycle of a new regime of chemotherapy with cyclophosphamide, vincristine sulfate, doxorubicin hydrochloride (adriamycin), and dexamethasone (R-Hyper CVAD). She had sequential lumbar punctures to reduce the high intracranial pressure. However, she developed neutropenic sepsis and refused the ventriculoperitoneal shunt.

During the last follow-up after two months, her visual acuity reduced to counting fingers in the right eye and only hand movement in the left eye. Her condition worsened, and she was planned for palliative care. Unfortunately, she passed away three months later.

## Discussion

BL derives its name from Dr. Burkitt who in 1958 reported mandibular malignancy in African children [4]. BL is a highly aggressive B-cell NHL and is one of the fastest-growing tumors in humans. It is characterized by the translocation and deregulation of the *MYC *gene on chromosome 8 with the potential to involve multiple organ systems. BL is characterized by inappropriately high levels of c-myc, which can result via several different mechanisms, most commonly by translocation of the long arm of chromosome 8 (containing the *MYC *gene) and the Ig heavy chain gene on chromosome 14. c-Myc overexpression leads to rapid B-cell proliferation, accounting for the rapid doubling time of BL tumor cells. Although most cases of BL have c-myc translocations, up to 10% of cases may be c-myc negative. Epstein-Barr virus, malaria arbovirus infection, as well as plant tumor promoters, are considered cofactors of the pathogenesis.

Symptoms and signs of BL depend on the site of involvement. Our patient had an initial presentation of persistent headaches with the subsequent blurring of vision in both eyes secondary to CNS involvement. Table 1 summarizes the cases of simultaneous bilateral optic nerve involvement in NHL. Our patient presented similarly to the majority of the cases in Table 1 with ocular involvement that developed later in the course of illness after diagnosis and initiation of treatment.

**Table 1:** Summary of cases with simultaneous bilateral optic nerve involvement in NHL. F: female; M: male; RE: right eye; LE: left eye; BE: both eyes; VA: visual acuity; CF: counting finger; HM: hand movement; NPL: no perception of light; OD: optic disc; HVF: Humphrey visual field; VF: visual field; CSF: cerebrospinal fluid; CNS: central nervous system; NHL: non-Hodgkin’s lymphoma

Patient	Age/Sex	Diagnosis	Ocular symptoms	Neurological symptoms	The onset of ON involvement	Ocular findings	Neuroimaging	CSF analysis	Treatment	Outcome
Dayan et al., 2000 [15]	74/F	Primary optic nerve low-grade B-cell NHL (diagnosis from optic nerve biopsy)	Intermittent visual obscuration for one year	Nil	Upon presentation of the disease	VA: RE 20/30, LE 20/80. Fundus: bilateral OD swelling. HVF: advanced bilateral VF constriction	Enlarged optic nerves (not enhanced)	Raised CSF protein level, mature lymphocytes	Low-dose radiotherapy to the optic nerves (total of 30 Gy to the optic nerves in 15 fractions over three weeks)	VA: RE 20/40, LE 20/80. General condition: well with no other systemic involvement
Kitzmann et al., 2008 [3]	39/M	Peripheral T-cell NHL, stage IV, with CNS involvement	Decreased vision in both eyes for four days	Nil	After two cycles of chemotherapy	VA: BE CF. Fundus: BE OD swelling with RE-associated flame-shaped hemorrhages	Mild enhancement of the right optic nerve and optic chiasm as well as leptomeningeal enhancement	Not done	Right optic nerve fenestration, chemotherapy, and radiotherapy (2,400 cGy)	VA: RE NPL, LE 2/200. General condition: died three months after his initial visual complaints
Zhu et al., 2015 [16]	68/F	Diffuse large B-cell lymphoma (DLBCL) in the anterior visual pathway	Bilateral progressive painless loss of vision for one year	Nil	Upon presentation of the disease	Details not mentioned	Suprasellar mass with significant homogenous enhancement and involvement of the optic chiasm, spreading along the two optic nerves and the right optic tract	LP not done	Tumor resection, radiotherapy (50 Gy in 25 fractions) and R-CHOP chemotherapy	VA: not mentioned. General condition: died 14 months after diagnosis
DeSouza et al., 2017 [17]	17/M	Burkitt lymphoma (post-renal transplant)	Left eye vision loss for six hours	Headache	Four months after diagnosis of Burkitt lymphoma	VA: RE 20/30, LE NPL. Fundus: bilateral blurred OD margins. The left OD had a large nasal hemorrhage and macular hemorrhages	Multifocal cortical and leptomeningeal CNS involvement of BL in the anteroinferior temporal lobes, parafalcine parietooccipital cortex, pineal gland, bilateral cerebellar hemispheres, and left optic nerve	Not mentioned	Whole-brain irradiation, 900 cGY over 12 fractions with systemic chemotherapy	VA: BE NPL. General condition: under treatment with pediatric oncology
Intan et al., 2019 [18]	19/F	Primary mediastinal B-cell NHL	Bilateral blurring of vision for one week	Headache, nausea, and vomiting	One year after diagnosis of lymphoma (patient default chemotherapy)	VA: BE 6/9. Fundus: bilateral OD swelling with disc hemorrhages and flame-shaped retinal hemorrhages	Cerebellar infiltration of lymphoma with generalized edema and obstructive hydrocephalus	Not mentioned	High-dose chemotherapy	VA: not mentioned. General condition: developed septicemia and multiorgan failure after two months
Intan et al., 2019 [18]	49/M	Left orbital NHL	Sudden onset of right eye blurred vision		Four years after remission	VA: RE 6/120, LE CF. Fundus: RE swollen OD with macula edema, dilated tortuous vein, and pre-retinal hemorrhages. LE: no fundus view due to mature cataract but B scan suggestive of LE OD swelling	Relapsed left orbital lymphoma with bilateral optic nerve infiltration	Not mentioned	Chemotherapy	VA: RE 6/36, LE not mentioned. General condition: achieved remission after three cycles of chemotherapy
Alsulami et al., 2021 [19]	62/F	Burkitt lymphoma with marrow involvement	Bilateral rapid painless decreased vision over one week	Nil	After three cycles of R-EPOCH	VA: RE 6/18, LE CF. Fundus: bilateral OD infiltration by a hemorrhagic white membrane with left eye vitritis	Abnormal enhancement involving both optic discs extending into the vitreous in the left eye	Flow cytometry revealed neoplastic cells consistent with Burkitt lymphoma	Whole-brain radiotherapy with a dose of 30 Gy in eight fractions	VA: no improvement. General condition: deteriorated rapidly and died
Present case	31/F	Burkitt lymphoma with marrow involvement	Left eye blurring of vision for one week	Severe headache with vomiting	After three cycles of DA-EPOCH-R	VA: RE 6/6, LE 6/45. Fundus: RE OD swelling, LE OD pallor	Increased contrast enhancement of the right optic nerve and increased leptomeningeal enhancement in the right frontal and temporal lobes	High CSF pressure 50 cmH_2_O. CSF analysis showed pleocytosis and the presence of lymphomatous infiltration	A serial lumbar puncture was done for symptomatic raised ICP. The chemotherapy regime was changed to R-Hyper CVAD	VA: RE CF, LE HM. General condition: deteriorated and died three months after the visual complaint

Secondary intraocular lymphoma is rare [5], and the most common ocular manifestation of this disease is non-granulomatous anterior uveitis and vitritis, with other orbital manifestations such as proptosis, periorbital swelling, and compressive optic neuropathy by paranasal sinus NHL [6]. These were not demonstrated in our patient. Visual functions are commonly preserved in the early stages of papilledema. In our patient, impaired optic nerve function including visual impairment with the evidence of optic disc swelling on the right and pallor on the left favored the diagnosis of infiltrative optic neuropathy. Direct infiltration, hematological, or CSF spread are among the possible pathway of optic nerve involvement [7]. In our patient, although headaches preceded the visual complaint, CNS infiltration with persistently raised ICP did not compromise the visual functions at the early stage.

Bone marrow biopsy is useful in diagnosing BL and determining bone marrow infiltration by BL. There is a typical “starry-sky” appearance in hematoxylin and eosin staining. Bone marrow biopsy of our patient upon presentation showed BL infiltration of the bone marrow, which is seen in about 30% of cases at presentation and confers an advanced stage of disease [8]. Imaging modalities such as computed tomography of thorax, abdomen, and pelvis (CT TAP)/positron emission tomography (PET) scan are needed to investigate for sites of involvement. A PET scan was not done for our patient due to unavailability. CT TAP in our patient revealed spleen involvement on top of CNS and optic nerve involvement. Apart from the leptomeningeal and optic nerve enhancement, as shown by the MRI in our patient, other radiological findings in CNS lymphoma include the enhancement of the subependymal layer, dura and cranial nerves, linear enhancement along perivascular spaces, communicating hydrocephalus, and lesions that are contrast-enhanced and isodense or hypodense in CT, hypointense or isointense on T1-weighted MRI, and isointense or hyperintense on T2-weighted MRI [9]. CSF analysis was negative for infection but positive for lymphomatous infiltration.

The mainstay of current treatment for BL is aggressive chemotherapy, and surgical treatment may not be indicated given improved chemotherapy and more local complication. Multi-regimen chemotherapy such as DA-EPOCH-R and R-Hyper CVAD (received by our patient) has up to 90% of remission rate [10]. BL is a highly aggressive but potentially curable disease. However, it is commonly associated with complications, such as tumor lysis syndrome, which can lead to multiorgan dysfunction [11]. Before CNS prophylaxis, there was a risk of 30-50% of CNS relapse in patients with BL [12]. The treatment regimens are prescribed in combination with high-dose methotrexate, high-dose cytarabine, and intrathecal chemotherapy [13]. Neurotoxicity secondary to chemotherapy such as vincristine-induced blindness has been reported in BL. Vincristine is a drug with a long half-life and extended vascular accumulation causing optic neuropathy secondary to optic nerve ischemia [14].

Although the most common prognostic factors in BL have not yet been identified, older age, advanced disease, poor performance status, and CNS or bone marrow involvement may lead to more unfavorable outcomes [13]. As for the prognosis, studies have shown that despite receiving combination therapy for those patients who had CNS involvement in lymphoma and developed visual symptoms later, the outcome was not favorable as death occurred within three weeks to two years after the onset of visual symptoms [7]. In addition, the visual prognosis was poor as reported by Ahle et al., with 71.4% having persistent poor vision or blindness [2]. Like the majority of cases in Table 1, our patient had poor visual outcomes with counting fingers in the right eye and hand movement in the left eye.

## Conclusions

BL is a highly aggressive B-cell lymphoma. It has a heterogeneous pattern of presentation and clinical course. Even with prompt initiation of therapy, patients with BL are at risk for early complications, including the optic nerve and CNS infiltration. CNS-directed therapies with a combination of intrathecal and high-dose systemic chemotherapy should be administered to patients with CNS involvement to deliver adequate CNS penetration and reduce the risk of CNS relapse.
